# Long March Toward Safe and Effective Analgesia by Enhancing Gene Expression of *Kcc2*: First Steps Taken

**DOI:** 10.3389/fnmol.2022.865600

**Published:** 2022-05-13

**Authors:** Wolfgang Liedtke

**Affiliations:** Regeneron Pharmaceuticals, Global Development Scientific Council, Tarrytown, NY, United States

**Keywords:** pain, spinal cord dorsal horn, KCC2, *Kcc2* gene expression enhancer, GSK3, delta-catenin, Kaiso

## Abstract

Low intraneuronal chloride in spinal cord dorsal horn pain relay neurons is critical for physiologic transmission of primary pain afferents because low intraneuronal chloride dictates whether GABA-ergic and glycin-ergic neurotransmission is inhibitory. If the neuronal chloride elevates to pathologic levels, then spinal cord primary pain relay becomes leaky and exhibits the behavioral hallmarks of pathologic pain, namely hypersensitivity and allodynia. Low chloride in spinal cord dorsal horn neurons is maintained by proper gene expression of *Kcc2* and sustained physiologic function of the KCC2 chloride extruding electroneutral transporter. Peripheral nerve injury and other forms of neural injury evoke greatly diminished *Kcc2* gene expression and subsequent corruption of inhibitory neurotransmission in the spinal cord dorsal horn, thus causing derailment of the gate function for pain. Here I review key discoveries that have helped us understand these fundamentals, and focus on recent insights relating to the discovery of *Kcc2* gene expression enhancing compounds *via* compound screens in neurons. One such study characterized the kinase inhibitor, kenpaullone, more in-depth, revealing its function as a robust and long-lasting analgesic in preclinical models of nerve injury and cancer bone pain, also elucidating its mechanism of action *via* GSK3β inhibition, diminishing delta-catenin phosphorylation, and facilitating its nuclear transfer and subsequent enhancement of *Kcc2* gene expression by de-repressing Kaiso epigenetic transcriptional regulator. Future directions re *Kcc2* gene expression enhancement are discussed, namely combination with other analgesics and analgesic methods, such as spinal cord stimulation and electroacupuncture, gene therapy, and leveraging *Kcc2* gene expression-enhancing nanomaterials.

Intracellular neuronal chloride is a critical determinant of sensory circuit integrity in the central nervous system (Ganguly et al., 2001; Woodin et al., 2003; Fiumelli and Woodin, 2007; Ben-Ari et al., 2012; Schulte et al., 2018). In post-developmental organisms of vertebrate and higher, this is dominantly a function of KCC2 which continuously extrudes chloride from essentially all CNS neurons (see Figure 1 for schematic; Rivera et al., 1999; Medina et al., 2014). This is certainly also true for neurons that function as the first central relay in any sensory circuit, including the primary gate in spinal cord and brain stem for afferents that carry an alarm signal, mainly for pain but also itch (Mantyh and Hunt, 2004; Price et al., 2005; Kahle et al., 2014a). Expression and function of KCC2 is thus aiding in the maintenance of a low chloride level within neurons (Figure 1). In turn, low intraneuronal chloride concentration is the critical pre-requisite for neurotransmission by GABA and glycine to function in an inhibitory manner (Delpire, 2000; Coull et al., 2003; Kahle et al., 2008, 2014a; Zeilhofer et al., 2012; Munro et al., 2013; Jaggi et al., 2015; Liang et al., 2015; Prescott, 2015). This is an absolutely critical prerequisite for normal postnatal CNS function. Weaker inhibition or even excitation in response to GABA—as during neural development—disempowers and corrupts mature neural circuits. In the spinal cord dorsal horn (SCDH) this has been associated with pathologic pain (Coull et al., 2003; Price et al., 2005; Austin and Delpire, 2011; Doyon et al., 2013; Medina et al., 2014; Come et al., 2019; Mapplebeck et al., 2019) and possibly also pathologic itch.

**FIGURE 1:**
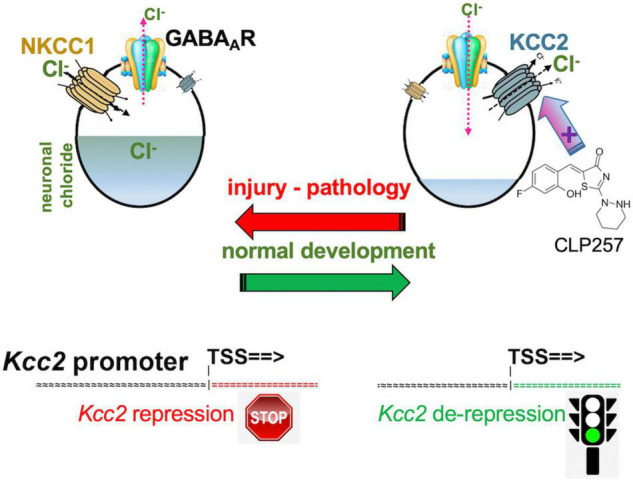
Developmental CNS chloride shift reversed in neural injury. Note the central role of the KCC2 chloride-extruding neuronal transporter and regulation of the *Kcc2* gene by de-repression. Compound CLP257 can enhance KCC2 chloride extrusion.

It remains to be understood whether attenuated gene expression of *Kcc2* and/or lack of function of KCC2 chloride extrusion causes pathologic pain. In preclinical mouse models, the answer is resoundingly affirmative. The conceptual loop has been closed and the initially articulated elegant concept has been robustly supported.

In a landmark discovery in 2003, Coull et al. (2003) from Yves DeKoninck’s group report that peripheral nerve injury leads to reduced expression of KCC2 in superficial layer neurons of the spinal cord, thus affecting pain behavior, namely hypersensitivity to peripheral stimuli, and spinal cord neurons’ synaptic anionic currents which were no longer inhibitory. In a follow-up paper, SCDH microglia were implicated in this pro-algesic plasticity mechanism (Coull et al., 2005). Of note, profoundly reduced expression of KCC2 was observed in superficial SCDH neurons. The authors conclude that “*the reported disruption of anionic homeostasis in lamina I neurons was sufficient to cause neuropathic pain*.”

The same group brought this exciting concept full circle by demonstrating that enhancing KCC2’s function in the setting of neuropathic pain, which was caused by peripheral nerve injury, was able to alleviate pain (Gagnon et al., 2013). A small molecule compound screen yielded KCC2 chloride extrusion enhancers. The top-pick compound, CLP257 (Figure 1), effectively treated neuropathic pain of nerve constriction injury, and also renormalized stimulus-evoked responses in pain gate SCDH neurons.

Yeo et al. (2021) very recently reported that enhancers of *Kcc2* gene expression also functioned as analgesics (see Figure 2 for schematic summary). Our discovery represents the direct correction of the initially observed defect by Coull et al. (2003) namely overcoming the lack of expression of *Kcc2* in SCDH superficial layer pain relay neurons by use of *Kcc2* gene expression-enhancing methods. We discovered two complementary approaches that enhanced *Kcc2* gene expression. Yeo et al. screened shelved cancer drugs to identify kenpaullone as their #1 pick because, amidst other “winners,” kenpaullone had a previous record of neuroprotection in various injury models, namely ALS cellular models with neurodegeneration, organismal ototoxicity with neurotoxicity to acoustic relay neurons, and generic neural injury (Skardelly et al., 2011; Yang et al., 2013; Liu et al., 2016; Teitz et al., 2018). We were particularly interested in interrogating cancer drugs because a sizeable number of them impact epigenetic regulation of genes to inhibit cell growth, thus increasing our chances of finding gene-expression enhancers for *Kcc2*. This rationale is based on previous observations that the *Kcc2* gene is critically regulated by epigenetic mechanisms (Yeo et al., 2013b; Yeo and Liedtke, 2020). In mouse preclinical pain models of nerve injury and cancer-associated bone pain, we found kenpaullone to be a safe and effective analgesic. The pain relief was profound, long-lasting, and with protracted onset, consistent with the drug having an impact on gene regulation. Derailed gene regulation underlies transition from acute to chronic pain (Okamoto et al., 2001; Wang et al., 2002; Hozumi et al., 2021; Morrison et al., 2021).

**FIGURE 2:**
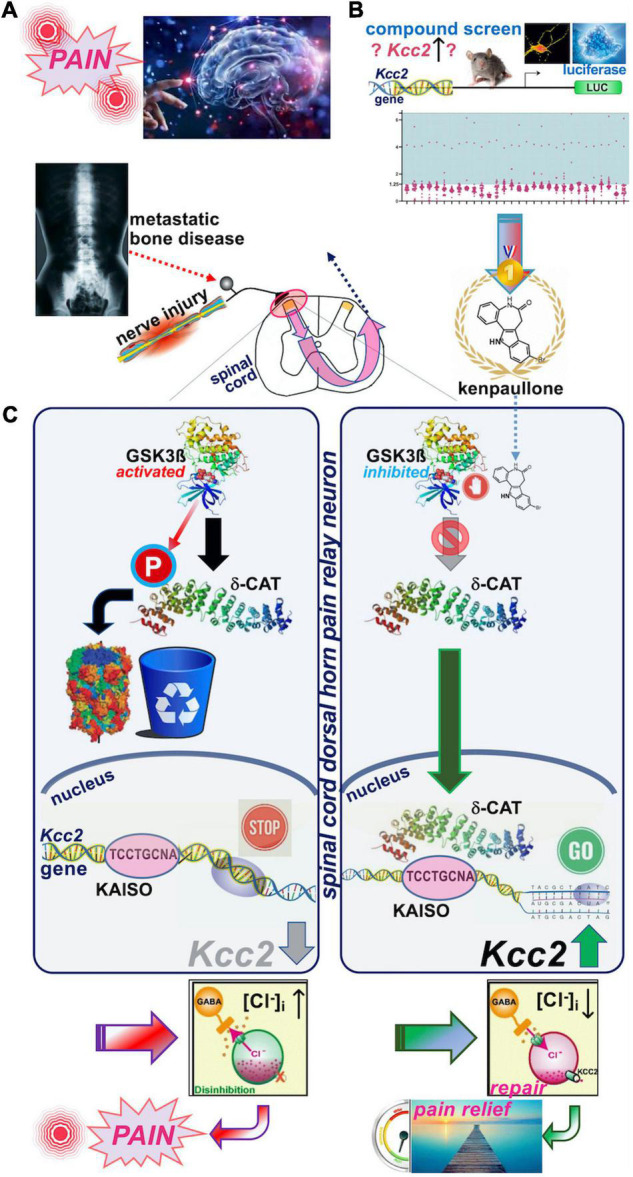
Schematic overview of the recent discovery of kenpaullone as *Kcc2* gene expression-enhancing compound. **(A)** Upper left. Nerve injury pain and bone cancer pain are serious and pressing unmet medical needs. Preclinical models were used to test kenpaullone, which proved highly effective in both. **(B)** Upper right. “Junkyard of cancer” drugs were screened for their potency to enhance *Kcc2* gene expression, using primary neurons derived from newborn mice cerebral cortexes. The *Kcc2* promoter, in these neurons, was driving luciferase (LUC), so that LUC could serve as a primary screening read-out. Kenpaullone was identified as a “winner,” capable of switching on the *Kcc2* gene, which previous research predicted to be beneficial for chronic pain. **(C)** Middle panels, left-hand: Nerve injury by constriction or cancer cells populating a bone activates GSK3β, an enzyme that tags other proteins with phosphate. In nerve cells dedicated to pain relay in the spinal cord, GSK3β tags delta-catenin (δ-CAT), which routs δ-CAT to the cellular garbage bin. Without δ-CAT in the cells’ nucleus, the *Kcc2* gene remains switched off. This in turn makes the pain relay neurons run full of chloride, which makes them electrically more jittery, with chronic “refractory” pain as a result. Right-hand panel: Treatment with kenpaullone inhibits GSK3β’s phosphate-tagging capability, so that δ-CAT becomes untagged, which clears the way to the nerve cells’ nucleus. There it binds to the DNA region of the *Kcc2* gene critical for switch-on or switch-off, the promoter. By binding there, δ-CAT reverts the switch-off to switch-on and the *Kcc2* gene runs again, making KCC2 protein. KCC2 in turn pumps chloride ions out of the pain-relay nerve cells, making them electrically more stable. This leads to circuit repair and pain relief, based on resetting of the genetic switches. Instead of Kenpaullone, δ-CAT can serve as payload of a gene therapy approach that directs expression of δ-CAT and hence KCC2 to pain relay nerve cells in the spinal cord.

We then tested whether kenpaullone affected GABA-evoked reversal potential as a direct function of chloride levels in SCHD pain-relaying neurons, and confirmed resoundingly that it did; it rendered the chloride reversal potential for GABA more negative, and thus electrically more stable (Yeo et al., 2021). In terms of the neurocellular mechanism of *Kcc2* gene expression-enhancing effects of kenpaullone, we indicted its GSK3β-inhibitory effects (Figure 2). In neurons, GSK3β phosphorylated delta-catenin at S259 (in rat; S267 is the human equivalent) and routs delta-catenin for cytoplasmic degradation. When selectively inhibiting GSK3β, non-phosphorylated delta-catenin trafficked to the neuronal nucleus where it enhanced *Kcc2* gene expression *via* KAISO transcription factor and two previously unrecognized Kaiso DNA binding sites which bracketed the *Kcc2* transcriptional start site. Using AAV9, delta-catenin viral transgenesis to spinal cord neurons was equally analgesic as kenpaullone, and it also enhanced *Kcc2 gene* expression in neural cells and the SCDH.

Additional *Kcc2* gene expression enhancing compounds were reported in a pioneering study by Tang et al. (2019) from Rudolf Jaenisch’s laboratory. They screened for enhancers of *Kcc2* gene expression in neuronalized human stem cells and identified GSK3β inhibitors as well, in addition to FLT3 kinase inhibitors and activators of sirtuin pathways and TRPV1. The latter three functional groups of compounds need to be assessed for their analgesic effects, and whether they can impact SCDH neuronal *Kcc2* gene expression, KCC2 chloride extrusion function, neuronal chloride, and E-GABA. For GSK3-inhibitors, identified by both Tang et al. (2019) and Yeo et al. (2021) the first attempt at elucidating their analgesic effects and mechanism-of-action as analgesics has been taken by Yeo et al. Additional GSK3-inhibitory molecules or *GSK3*β gene knockdown await future interrogation. FLT3-inhibitors have already been implicated as analgesics, yet with postulated mechanism of action in peripheral neurons (Rivat et al., 2018). Activation of sirtuin pathways and TRPV1 might be less relevant for impacting *Kcc2* gene expression in SCDH pain relay neurons, yet molecules and approaches impacting these pathways also await dedicated experimental examination.

*Kcc2* gene expression is fundamental to all functions of the KCC2 protein, and its attenuation has been demonstrated to be causal and key for chronic pain, with a clean demonstration of causality in several preclinical models of pathologic pain, as detailed above. In addition, there is suggestive evidence in human spinal circuit models (Dedek et al., 2019), a more recent experimental platform based on early post-mortem spinal cords.

Chloride-extruding function of KCC2 can be impaired by post-translational modification. KCC2 phosphorylation has been demonstrated to impact neuronal excitability, having possible disease relevance for epilepsy, with key discoveries made by several investigators, amongst them the groups of Kristofer Kahle, Stephen Moss, Eric Delpire, and other investigators with distinguished records (Strange et al., 2000; Woo et al., 2002; Zhu et al., 2008; Lee et al., 2010, 2011; Kahle et al., 2013, 2014b, 2016a; Kelley et al., 2018; Pisella et al., 2019; Watanabe et al., 2019; Kontou et al., 2021). Their conclusions coalesce to a concept of enhancing KCC2 chloride extrusion function by inhibiting its phosphorylation, aiming to better treat refractory epilepsy. There is also some suggestive evidence that WNK kinase has relevance for pathologic pain with postulated mechanism-of-action *via* KCC2 phosphorylation, which renders it a less effective transporter (Kahle et al., 2016b). However, what has to be demonstrated at increased resolution, with strict focus on SCDH pain relay neurons, is whether analgesic effects of WNK-inhibition or knockdown rely on critical proalgesic function of WNK as it phosphorylates KCC2 in these neurons, subsequently causing elevated intraneuronal chloride. This in turn translates to more positive, thus electrically less stable E-GABA.

Re gene regulation of *Kcc2*, the following gene regulatory elements have been discovered in *Kcc2* gene regulatory DNA sequences (see Figure 3; reviewed in Yeo and Liedtke, 2020, see this chapter for more detailed account):

**FIGURE 3:**
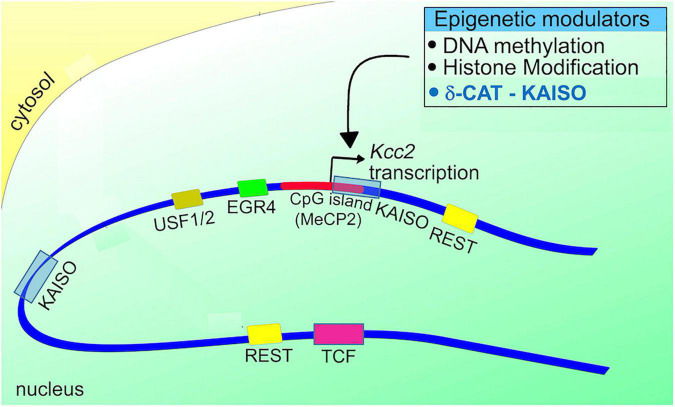
Schematic representation of the *Kcc2* promoter. Note the positioning of DNA binding sites for relevant transcriptional modulators in relation to the *Kcc2* transcriptional start site. The connection to epigenetic regulation, suggested since the discovery of *Kcc2* gene expression regulated by REST-RE-1 de-repression, is further enhanced by the impact of the CpG island surrounding the *Kcc2* transcriptional start site, and the two Kaiso DNA binding sites “bracketing” the *Kcc2* transcriptional start site. Since Kaiso-KAISO interactions, which by default are repressive, rely on DNA methylation, the delta-catenin—Kaiso interaction, as recently demonstrated in Yeo et al. (2021) is referenced as an epigenetic modulator.

(1) dual RE-1 sites for binding of the REST complex, as described in Yeo et al. (2009).
(2) Egr consensus binding site for binding of EGR4 (Uvarov et al., 2006).
(3) E-box element for binding of USF1/2 (Medina et al., 2014; Yeo and Liedtke, 2020).
(4) dual Kaiso binding sites for binding of KAISO and delta-catenin (Yeo et al., 2021).
(5) CpG island surrounding the transcriptional start site which can subserve DNA methylation-mediated repression, first described in Yeo et al. (2013a).

Of these, only Kaiso has been found to be involved in regulation of *Kcc2* in the context of pain (please see above synopsis of Yeo et al., 2021). Importantly, we have to remember that Kaiso is a transcriptional repressor, its repressive function dependent on DNA methylation, and that Kaiso interacting with delta-catenin can change the binding of this complex to regulatory DNA sequences, and also the directionality of transcriptional regulation (Prokhortchouk et al., 2001; Lopes et al., 2008; Dai et al., 2011; Kaplun et al., 2021). This means that repression can change into de-repression. In the referenced (Yeo et al., 2021) study we discovered exactly that, with a net result of GSK3β inhibition leading to enhanced *Kcc2* gene expression *via* a mechanism of GSK3β ==> delta-catenin ==> delta-catenin/KAISO ==> Kaiso (*Kcc2*-promoter), which de-repressed *Kcc2*. Our data suggest that this mechanism of GSK3β inhibitory effect of kenpaullone, also non-phosphorylated delta-catenin viral transgenesis to SCDH neurons, evoked enhancement of *Kcc2* gene expression in neurons and analgesia in live animals.

Relevance for pain of the other transcriptionally relevant DNA binding sites and other transcriptional and epigenetic mechanisms of the *Kcc2* gene appears plausible and possible. However, the contribution of such *Kcc2* gene-regulatory mechanisms awaits future experimental confirmation in pain-relevant preclinical models.

The recent Yeo et al. (2021) study measured chloride reversal potential in response to GABA (E-GABA) in lamina-II SCDH neurons. Also recently, DeKoninck’s group made an important contribution to the improved understanding of the cellular architecture and regional physiologic differences within the SCDH (Ferrini et al., 2020). Lamina-I and lamina-II (external) receive peptidergic thermal nociceptive afferents, while lamina-II (internal) receives mechanical nociceptive afferents. Physiologically, *Kcc2* expression is more robust in lamina-II (internal) with resulting lower intraneuronal chloride and “inhibitory robustness.” This finding indicates that injury-mediated attenuated expression of *Kcc2* can derail the inhibitory robustness of SCDH noci-transmission more effectively, perturbing the primary pain gate with increased impact. This could contribute to the clinically more dominant effect of mechanical allodynia in neuropathic pain vs. thermal allodynia. The Ferrini et al. (2020) study also reiterates the need to learn more about molecular and physiologic identity of SCDH neurons. So far, we know that there are tachykinin-expressing and somatostatin-expressing lineages, both with relevance for pain (Gamse et al., 1981; Morton et al., 1989; Sandkuhler et al., 1990; Yin, 1995; Lagraize et al., 2010; Shi et al., 2014; Gutierrez-Mecinas et al., 2017; Chamessian et al., 2018). There likely are more lineages. The powerful method of single-cell RNA-seq (scRNAseq) will very likely move this field forward in non-incremental steps.

A critical question arises: how does the injury response differ in these neurons, if it does? And in particular, this question refers to attenuation of *Kcc2* gene expression and impaired chloride extrusion function of residual KCC2 transporter protein in these specific SCDH neuronal lineages. If a defined neuronal phenotype spreads across laminae, then lamina-specificity becomes yet another relevant criterion to take into account.

And certainly, we are in need of elucidating any new insights relating to their possible sex- and age-specificity (Mapplebeck et al., 2019).

Yet another deeper dive into neuronal network excitability and its reliance on neuronal chloride and KCC2 is worth discussing here. Kirmse et al. (2015) report that, in the early postnatal occipital cortex of the mouse (Kirmse et al., 2015), GABA is a predominantly depolarizing agent in neurons at p3-4, yet has an inhibitory function at the neuronal network level. The impact of the enhancement of *Kcc2* gene expression or KCC2 chloride extrusion function was not examined in this paper. In a related study, Otsu et al. (2020) report their findings in adult mouse hippocampal pyramidal and parvalbumin^+^ neurons, examined by e-phys in slice culture. They found activation of GABA-A receptors to be depolarizing at the cellular level of both neuronal lineages, yet inhibitory at the circuit level. Inhibiting KCC2 chloride extrusion further depolarized the neurons and brought resting potential closer to the action potential threshold, promoting firing. Inhibition of KCC2 function therefore rendered the interrogated hippocampal network more excitable, in keeping with findings of pain relay neurons in the SCDH. Beyond this similarity, these findings of GABA depolarizing at neuro-cellular level, *via* shunting, yet inhibitory at network level (Kirmse et al., 2015; Otsu et al., 2020), can perhaps help us better understand hyperexcitability of SCDH pain relay neurons, as it relates to their response to GABA and glycine in spinal cord slices, but awaits further study relating to circuit function as it contributes to the clearly present behavioral correlates of pain.

Whereas the neural organization of pain transducing and transmitting structures is principally similar for DRG- and trigeminal/cranial nerve-mediated pain, there could be some critical differences, namely circuitry that underlies specific clinical features such as higher emotional impact of trigeminally-mediated pain (Rodriguez et al., 2017) or cellular-physiologic and/or circuit mechanisms that underlie different pharmacologic profiles, such as triptans not effective against DRG-mediated pain (Moskowitz, 1993; Diener and Limmroth, 1999). The ask for future studies will be to verify any mechanism discovered in DRG-mediated pain in the trigeminal system. Previously conducted studies suggested *Kcc2* downregulation in response to injury in trigeminal pain relay neurons (Wu et al., 2009; Wei et al., 2013), but one recent noteworthy investigation failed to document this for the commonly used infraorbital nerve constriction model (Castro et al., 2017).

In clinics, refractory severe migraines are effectively treated with prochlorperazine (i.v) (Friedman et al., 2017; Cook and Newberry, 2018), a seasoned phenothiazine drug which was identified in a repurposing screen to enhance KCC2 chloride extrusion function (Liabeuf et al., 2017). This perhaps indicates migraine pain-relevant KCC2 expression and function in trigeminal pain relay circuits.

It will be interesting to examine the trigeminal pain circuit that originates in primary afferents of trigeminal-specific monosynaptic projections from trigeminal ganglion to the lateral parabrachial nucleus (Rodriguez et al., 2017). Also interesting to discover is whether KCC2 expression and function contribute to pathologic pain as it transmits through this recently discovered circuit, which is directly plugged into the emotional processing centers of the brain. This recently discovered and previously unknown neural connectivity is considered to underlie strong negative emotional connotation of trigeminal pain.

Boosting expression and/or function of KCC2 appears a naturally synergistic approach to many analgesic methods (Figure 4). Enhancement of *Kcc2* gene expression in a form of renormalization of its expression may conceptually have a higher appeal because it will be less prone to tachyphylaxy, as it targets longer-term gene regulation rather than short-term regulation of the effector protein (Yeo et al., 2021). Enhancing the rate of chloride extrusion beyond the physiologic rate might quickly exhaust the transporter, an effect of insufficient duration for treatment of chronic pathologic pain.

**FIGURE 4:**
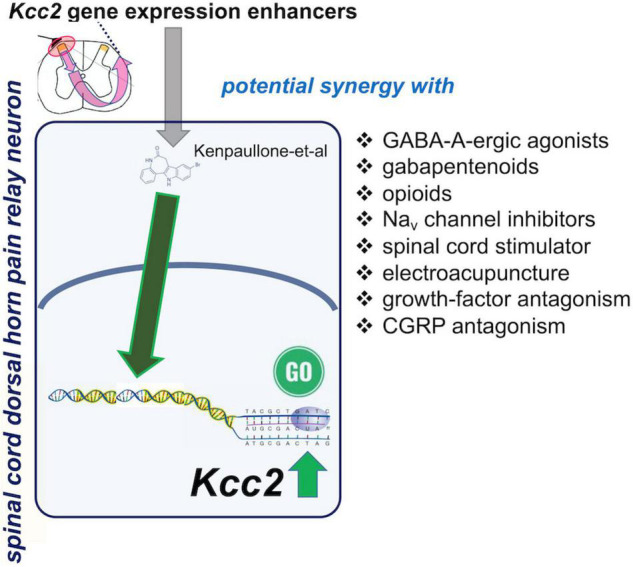
Spinal cord dorsal horn pain relay neuron, site of action of *Kcc2* gene expression-enhancing treatments: possible synergies with other analgesic treatments.

Chronic pathologic pain typically needs to be treated with a combination of treatments because no one single treatment can “magically” rectify the maladaptive plasticity of chronic pain. Under a premise of a polypharmacy-polytherapy for chronic pathologic pain, enhancement of *Kcc2* gene expression might be a winning combination with GABA-A agonistic compounds (Figure 4). This has already been demonstrated for chloride extrusion enhancement in preclinical models by DeKoninck’s group (Lorenzo et al., 2020). The medication typically prescribed by practitioners is clonazepam, with its empirically known improved effectiveness against trigeminal pain (Smirne and Scarlato, 1977). Another class of medications likely benefitting from combination with a *Kcc2* gene expression enhancer is opioids, which could be reduced to safer levels. The near future will likely see enhanced opioid-derived therapeutics which are safer than “old-fashioned” opioids (one representative example; Xu et al., 2020). Inhibitory compounds have been developed to target the Nav1.7 channel, a target strongly indicted by genetic evidence from humans and preclinical model systems (Xiao et al., 2010; King and Vetter, 2014; Berta et al., 2017; Zhang and Gan, 2017). Up to this day, however, results with these Nav1.7-targeting approaches have been sobering (Mulcahy et al., 2019). Combination with *Kcc2* gene expression-enhancing therapies could represent the breakthrough needed to salvage this situation—unless Nav1.7 is, for a yet unknown reason, principally non-translatable.

Another unexpected difficulty during recent developments of new analgesic approaches has been encountered when using NGF-neutralizing therapeutic monoclonal antibodies. With these, therapeutic effectiveness against severe osteoarthritis pain was observed, although in some patients it was also associated with pathologic bone remodeling of periarticular bones (Hochberg, 2015; Miller et al., 2017). This has led the FDA and its European counterpart to vote against approval of tanezumab, one such anti-NGF monoclonal antibody, for osteoarthritis pain. A solution might be to combine anti-NGF at a low dose where bone remodeling is not a part of the effects, with *Kcc2* gene expression-enhancing strategies.

Analgesic approaches also comprise neurostimulation/neuromodulation, which have found a niche in a narrow segment of select patients. Generally, neurostimulation has been hindered by the waning of effectiveness over time, thus lacking long-term effects (Hayek et al., 2015). Recent progress has been made with so-called closed loop neurostimulation systems, such as for spinal cord stimulation (Mekhail et al., 2022), but it is not clear whether this updated stimulation protocol is resistant to losing its effectiveness over time (Noorsal et al., 2021; Pilitsis et al., 2021). It is tempting to envision use of neurostimulators jointly with *Kcc2* gene expression-enhancing strategies so that neuronal chloride in pain circuits remains low with resulting persistent inhibitory robustness—hopefully locking in the analgesic benefits of the neurostimluation methods more long-term.

A related analgesic method is electroacupuncture, which has been associated with enhanced KCC2 function (Li et al., 2018; Yuan et al., 2022). Combination with *Kcc2* gene expression-enhancing methods might extend effectiveness of electroacupuncture over time.

Yeo et al. (2021) also report analgesia as a result of intrathecal injection of AAV9 with cargo of delta-catenin (human isoform) with a S276A mutation to prevent phosphorylation by GSK3β, the delta-catenin transgene driven by the human synapsin promoter. We showed early and robust expression in SCDH neurons, using this method, and relatively sparse and delayed expression in DRGs. This transgene functioned as *Kcc2* gene expression-enhancer in neural cells and in spinal cord dorsal horn. Further translational development appears readily feasible (Figure 5), namely the use of more selective AAV capsids for targeting SCDH neurons and improved promoter for robust long-term expression. This could form the basis for ultra-long-term renormalization of defective *Kcc2* expression in the SCDH as a result of multiple forms of neural injury, namely peripheral mechanical, inflammatory or chemo-toxic injury, other forms of tissue injury, injury by malignant cell growth or therapy thereof, spinal cord injury, and other pain-causing injuries.

**FIGURE 5:**
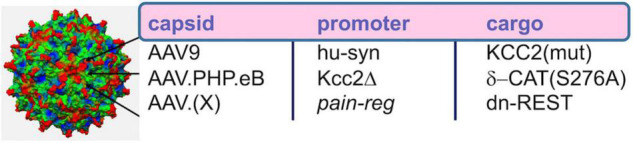
AAV-based gene therapeutic avenues toward enhancement of *Kcc2* gene expression. Re capsid type, AAV9 worked well in Yeo et al., where intrathecal injection led to viral transduction of central neurons in the spinal cord. AAV.PHP.eB might have increased tropism for central neurons over peripheral neurons, and AAV.(X) refers to future capsid variants that will ideally target pain relay neurons selectively. Re promoter, the short human synapsin promoter, was used successfully in Yeo et al. Kcc2Δ refers to an engineered *Kcc2* promoter that has been cleared of repressor sites, such as those lacking both RE-1 elements and lacking the upstream Kaiso site; this promoter will have to be tested in future studies. Re cargo, this refers to transgene. This could be KCC2 itself. In that case, point mutations that enhance KCC2 chloride extrusion will be advantageous. For indirect enhancement of gene expression which might be more suitable for long-term effective treatments, delta-catenin that cannot be phosphorylated or dominant-negative REST are suitable options.

In keeping with the new concept of enhancing *Kcc2* gene expression by an upstream enhancing molecule, delta-catenin, an alternative method of spinal viral transgenesis was described by Hui-lin Pan’s group, who directly overexpressed a *Kcc2* transgene targeting spinal and DRG neurons, using lentivirus (Li et al., 2016). They observed long-lasting analgesia, and likely contribution by both CNS and PNS pain circuit neurons. The individual contributions of these lineages to the analgesic effect need to be untethered, and methods on how to prevent long-term silencing of transgene expression need to be developed.

Enhancement of *Kcc2* gene expression can also be accomplished by leveraging a material science-based finding from my former laboratory (Figure 6). Liedtke et al. (2013) reported enhanced *Kcc2* gene expression in CNS neurons and cortical neuronal cultures by interfacing the neurons with electrically conductive few-walled carbon nanotube (fwCNT) matrix (Figure 6A; Liedtke et al., 2013). Biocompatible polymer biofilms [PGSA (biodegradable), PDMS (non-biodegradable)], with regularly patterned cone-shaped multiplex surface, similar to microneedles, with indentation depth of 200 μm, can be coated with fwCNT (Figures 6B,C). The fwCNT that coats the insertional devices can be functionalized with compounds that enhance *Kcc2* gene expression further, or other effective analgesic compounds. Then, *via* microsurgery, these devices are interfaced with the spinal cord dorsal horn, and inserted to maximal insertion depth (Figure 6D). Preclinical use in rats, and then in non-rodent translational pain preclinical models, will inform us about the suitability and translational effectiveness of this approach, a “neuroprosthetics-2.0” method, to combat severe pathologic pain.

**FIGURE 6:**
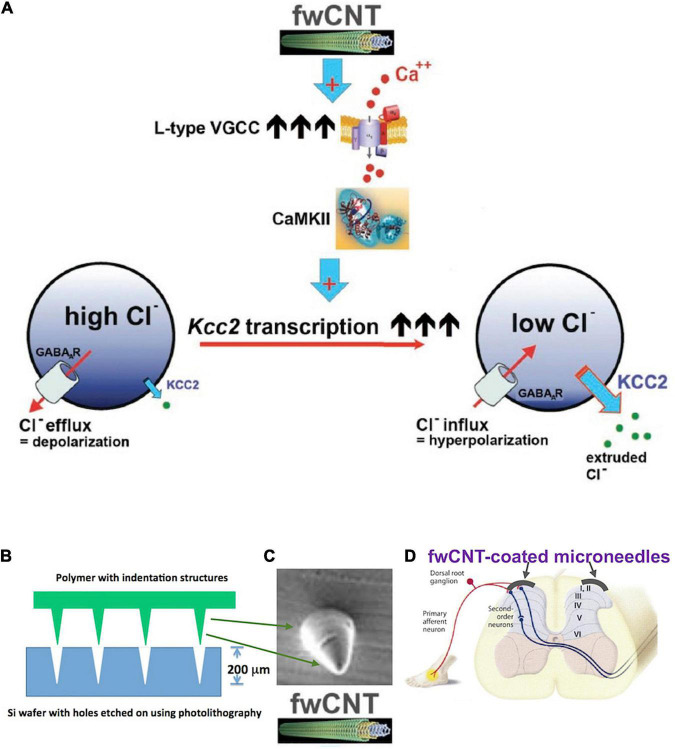
*Kcc2* gene expression-enhancement based on few-walled carbon nanotubes for coating of microneedle-related insertional devices, for insertion into the spinal cord dorsal horn. **(A)** Summary figure of a previous study by Liedtke et al. (2013) that drives home the point of electrically conductive few-walled carbon nanotubes (fwCNT) robustly enhancing *Kcc2* gene expression, *via* a mechanism of calcium-influx mediated activation of CaMKII. **(B)** A future neuroprosthetic device for enhancement of *Kcc2* gene expression can be fabricated with approximate insertion depth of 200 μm, using biodegradable (or non-degradable, yet biocompatible) polymers and microtechnology fabrication methods. **(C)** Cone-shaped single needle at 200 μm height, intended for microneedle format (array density at 50–100/cm), for subsequent coating with fwCNT. **(D)** Suggested insertion of the fwCNT-coated microneedle device into the spinal cord dorsal horn.

Almost two decades after DeKoninck’s founding discovery of attenuated expression of *Kcc2* in the primary pain gate of the spinal cord and resulting hyperexcitable GABA-ergic anionic neurotransmission, as a result of peripheral nerve injury, research into mechanisms and clinical translation to repair this fundamental defect has come a long way, as summarized here. We have taken the first steps. However, our march toward novel rationally targeted analgesics has to continue; we have to keep walking to translate these insights into clinical reality.

## Author Contributions

WL wrote the manuscript and completely conceptualized it.

## Conflict of Interest

WL is a full-time executive employee of Regeneron Pharmaceuticals, Tarrytown, NY, United States.

## Publisher’s Note

All claims expressed in this article are solely those of the authors and do not necessarily represent those of their affiliated organizations, or those of the publisher, the editors and the reviewers. Any product that may be evaluated in this article, or claim that may be made by its manufacturer, is not guaranteed or endorsed by the publisher.
