# Erratum: Long March Toward Safe and Effective Analgesia by Enhancing Gene Expression of *Kcc2*: First Steps Taken

**DOI:** 10.3389/fnmol.2022.979385

**Published:** 2022-07-05

**Authors:** Frontiers Production Office

**Affiliations:** Frontiers Media SA, Lausanne, Switzerland

**Keywords:** pain, spinal cord dorsal horn, KCC2, *Kcc2* gene expression enhancer, GSK3, delta-catenin, Kaiso

Due to a production error, the “Conflict of Interest” statement incorrectly stated that “WL was a full-time” instead of “WL is a full-time.”

The corrected section should read:

“WL is a full-time executive employee of Regeneron Pharmaceuticals, Tarrytown, NY, United States.”

The publisher apologizes for this mistake. The original version of this article has been updated.

